# Ocellatin peptides from the skin secretion of the South American frog *Leptodactylus labyrinthicus* (Leptodactylidae): characterization, antimicrobial activities and membrane interactions

**DOI:** 10.1186/s40409-017-0094-y

**Published:** 2017-01-19

**Authors:** Karla A. G. Gusmão, Daniel M. dos Santos, Virgílio M. Santos, María Esperanza Cortés, Pablo V. M. Reis, Vera L. Santos, Dorila Piló-Veloso, Rodrigo M. Verly, Maria Elena de Lima, Jarbas M. Resende

**Affiliations:** 1 Departamento de Química, Instituto de Ciências Exatas, Universidade Federal de Minas Gerais (UFMG), Belo Horizonte, MG Brazil; 2 Instituto de Engenharia, Ciência e Tecnologia, Universidade Federal dos Vales do Jequitinhonha e Mucuri (UFVJM), Janaúba, MG Brazil; 3 Departamento de Bioquímica e Imunologia, Instituto de Ciências Biológicas, Universidade Federal de Minas Gerais (UFMG), Belo Horizonte, MG Brazil; 4 Departamento de Odontologia Restauradora, Faculdade de Odontologia, Universidade Federal de Minas Gerais, Belo Horizonte, MG Brazil; 5 Departamento de Microbiologia, Instituto de Ciências Biológicas, Universidade Federal de Minas Gerais (UFMG), Belo Horizonte, MG Brazil; 6 Departamento de Química, Universidade Federal dos Vales do Jequitinhonha e Mucuri (UFVJM), Diamantina, MG Brazil

**Keywords:** *Leptodactylus labyrinthicus*, Ocellatins, Antimicrobial peptides, Peptide membrane interactions

## Abstract

**Background:**

The availability of antimicrobial peptides from several different natural sources has opened an avenue for the discovery of new biologically active molecules. To the best of our knowledge, only two peptides isolated from the frog *Leptodactylus labyrinthicus*, namely pentadactylin and ocellatin-F1, have shown antimicrobial activities. Therefore, in order to explore the antimicrobial potential of this species, we have investigated the biological activities and membrane interactions of three peptides isolated from the anuran skin secretion.

**Methods:**

Three peptide primary structures were determined by automated Edman degradation. These sequences were prepared by solid-phase synthesis and submitted to activity assays against gram-positive and gram-negative bacteria and against two fungal strains. The hemolytic properties of the peptides were also investigated in assays with rabbit blood erythrocytes. The conformational preferences of the peptides and their membrane interactions have been investigated by circular dichroism spectroscopy and liposome dye release assays.

**Results:**

The amino acid compositions of three ocellatins were determined and the sequences exhibit 100% homology for the first 22 residues (ocellatin-LB1 sequence). Ocellatin-LB2 carries an extra Asn residue and ocellatin-F1 extra Asn-Lys-Leu residues at C-terminus. Ocellatin-F1 presents a stronger antibiotic potential and a broader spectrum of activities compared to the other peptides. The membrane interactions and pore formation capacities of the peptides correlate directly with their antimicrobial activities, i.e., ocellatin-F1 > ocellatin-LB1 > ocellatin-LB2. All peptides acquire high helical contents in membrane environments. However, ocellatin-F1 shows in average stronger helical propensities.

**Conclusions:**

The obtained results indicate that the three extra amino acid residues at the ocellatin-F1 C-terminus play an important role in promoting stronger peptide-membrane interactions and antimicrobial properties. The extra Asn-23 residue present in ocellatin-LB2 sequence seems to decrease its antimicrobial potential and the strength of the peptide-membrane interactions.

**Electronic supplementary material:**

The online version of this article (doi:10.1186/s40409-017-0094-y) contains supplementary material, which is available to authorized users.

## Background

The resistance acquired by pathogens against the commonly used antibiotics has become an important health issue throughout the globe and, therefore, the discovery of new drugs is a topic of discussion in the scientific community. Antimicrobial peptides (AMPs) have emerged as an interesting option, since these compounds usually present broad spectra of activities against several microorganisms, including bacteria, fungi and viruses [1, 2]. These characteristics have therefore stimulated the isolation, as well as the characterization and antimicrobial activity evaluation of numerous of these compounds, and nowadays thousands of sequences can be found in the databanks [3]. In particular, a huge variety of active peptides is found in the skin secretion of anurans (frogs and toads) and many of these substances play very important roles in the immune systems of these animals, acting as a first line of defense against microorganisms [4–6]. AMPs from anurans are usually composed of 10 to 48 amino acid residues and they present a variety of different structural motifs, although many of them are usually cationic and present amphipathic helix conformations [4, 7].

It is well known that the membrane-interaction process is a key step for the antimicrobial activity of these compounds, which may promote membrane lyses, as described by several models [8], although some peptides seem to interact with internal targets after crossing the bilayer barrier [9–11]. Therefore, the understanding of the mechanism of action demands biophysical investigations of these compounds in membrane mimetic environments, which can be performed by different techniques, such as isothermal titration calorimetry, circular dichroism and nuclear magnetic resonance spectroscopies, among others [12–15]. The therapeutic potential of AMPs is sometimes limited by low selectivity issues, in cases where the peptide also presents toxicity against eukaryotic cells. Therefore, several membrane models, such as vesicles containing different lipid compositions, including cholesterol, can be employed to investigate the selectivity of these compounds [16, 17].

In recent decades, antimicrobial peptides have been isolated from from anurans, including those from the *Leptodactylus* genus that contains about 75 species [4, 18]. These animals are found in South America, especially in Brazil and the Antilles [19–24]. Although peptide sequences from several anuran species have already been reported in the literature, some species have been poorly or not investigated at all up to now. To the best of our knowledge, only two peptides isolated from the frog *Leptodactylus labyrinthicus*, namely pentadactylin and ocellatin-F1, have shown antimicrobial activities.

The compound pentadactylin, having been isolated from the frog species *Leptodactylus labyrinthicus*, has also presented anticancer activity and nontoxicity against erythrocytes [25]. Oscillatin-F1, an antimicrobial peptide that was originally found in the skin secretion of the mountain chicken frog *Leptodactylus fallax* [24], has also been recently isolated from the skin secretion of *Leptodactylus labyrinthicus* by Cunha Neto et al. [26]. In order to further explore the biological potential of the *Leptodactylus labyrinthicus* skin secretion, we present here the biological characterization of three peptides isolated from the skin secretion of this frog species. We have also investigated the interaction of these peptides with different membrane mimetic systems, such as zwitterionic and anionic detergent micelles and phospholipid bilayers by using different biophysical approaches.

## Methods

### Materials

1-palmitoyl-2-oleoyl-*sn*-glycero-3-phosphocholine (POPC), 1-palmitoyl-2-oleoyl-*sn*-glycero-3-phospho-L-serine (POPG) and dodecylphosphocholine (DPC) were purchased from Avanti Polar Lipids (USA). Rink amide polystyrene resin and amino acid derivatives for peptide synthesis were from Iris Biotech GmbH (Germany); trifluoroacetic acid (TFA, analytical and HPLC grades), triisopropylsilane and 2,2,2-trifluoroethanol from Sigma-Aldrich (USA); *N*,*N*’-diisopropylcarbodiimide from Fluka (Germany); 1-hydroxybenzotriazole and 1,2-etanoditiol from Nova Biochem-Merck (Germany); *N*,*N*-dimethylformamide, diisopropyl ether, chloroform and dichloromethane were obtained from Vetec (Brazil); and acetonitrile (HPLC grade) from JT Baker (USA). Sodium dodecyl sulfate (SDS), calcein, Sephadex® G-50 medium, Triton X-100, and HEPES from Sigma-Aldrich (USA). Unless stated otherwise analytical grade solvents were used.

### Peptide purification

The *L. labyrinthicus* skin secretions were obtained by scraping the dorsum of the frog and then diluted in Milli-Q water, lyophilized and kept frozen at −80 °C for subsequent use. Aliquots of lyophilized skin secretion were dissolved in 0.1% (v/v) TFA/water, filtered (0.22 μm) and centrifuged at 10,000 rpm at 4 °C for 10 min. The supernatant was purified on a C8 reversed-phase column (Discovery Supelco, 4.6 × 250 mm). Elution was performed with a gradient of acetonitrile containing TFA 0.1% (solvent B) at a flow rate of 1 mL.min^−1^ (0–10 min, 0% B; 10–16 min, gradient of 0–20% B; 16–100 min, 20–65% B; 100–108 min, 65–100% B; 108–116 min, 100% B, 116–117 min, 100–0% B and 117–125 min, 0% B). The experiments were monitored at 214 nm and the fractions were collected and lyophilized.

### Mass fingerprinting MALDI-ToF/ToFMS

The fractions obtained from the *L. labyrinthicus* skin secretion by chromatographic separation were analyzed by mass spectrometry performed on a MALDI-ToF/ToF mass spectrometer (Autoflex™ III SmartBeam spectrometer, Bruker Daltonics, Germany) in linear and reflector modes and the spectra were processed with MassLynx^TM^3.5 (UK) and FlexAnalysis 3.3 (Bruker Daltonics, Germany).

Briefly, solubilized fractions (0.5 μL of sample, variable concentrations) were spotted onto the target followed by 0.5 μL of CHCA (α-cyano-4-hidroxycinnamic acid) or DHB (2,5-dihydroxybenzoic acid) matrix solution (60% acetonitrile/0.3% TFA), and allowed to dry at room temperature (dried-droplet method). Peptide Calibration Standard II (700–4000 Da) and Protein Calibration Standard I (3000–25,000 Da) (Bruker Daltonics, Germany) were used as external calibration standards. Mass spectra from the average of 256 laser pulses from m/z 600 to 39,400 were obtained.

### Amino acid sequencing

The primary structures of the purified peptides were determined by automated Edman degradation (PPSQ-21A protein sequencer, Shimadzu, Japan) coupled to reversed-phase separation of the PTH-amino acids on a WAKOSIL-PTH column (4.6 mm × 9250 mm) (Wako, Japan).

### Peptide synthesis, purification and characterization

The peptides, with amidated C-terminus, were prepared by solid-phase synthesis on a Rink amide resin by using the Fmoc strategy [27]. Couplings were performed with *N*,*N*’-diisopropylcarbodiimide/1-hydroxybenzotriazole in *N*,*N*-dimethylformamide for 120 min under stirring (240 rpm). Cleavage and final deprotection were conducted with TFA:triisopropylsilane:ethanedithiol:water (94.0:1.0:2.5:2.5, v:v:v:v) for 180 min at room temperature. The peptide products were precipitated with diisopropyl ether, extracted with water and lyophilized. Then, peptides were purified by RP-HPLC (Varian Pro Star 210 Series, USA) using a preparative C18 column (Vydac C18, 300 × 7.8 mm, USA) eluted by a linear gradient of acetonitrile containing TFA 0.1% (solvent B) (0–5 min, a gradient of 20–35% acetonitrile in 0.1% TFA in water; 5–20 min, gradient of 35–45% acetonitrile containing 0.1% TFA in water; 20–35 min, 45-100% acetonitrile containing 0.1% TFA in water; 35–37 min, 100% acetonitrile with 0.1% TFA; 37–40 min, 100–20% acetonitrile containing 0.1% TFA in water). A flow of 2.0 mL.min^−1^ was used and the peptides were detected at 214 nm. The identities of the peptides were confirmed by MALDI-ToF/ToF mass spectrometry (autoflex™ III SmartBeam spectrometer, Bruker Daltonics, Germany).

### Vesicle preparation

The correct amount of POPC or POPC:POPG (3:1 mol:mol) was firstly dissolved in chloroform and the solvent was removed with a rotary evaporator resulting in a thin film, which was further dried under vacuum to remove the residual solvent. The film was then hydrated with ultra-pure H_2_O and vortex-stirred leading to the formation of large multilamellar vesicles (LMVs). Large unilamellar vesicles (LUVs) were obtained by submitting the suspension to five cycles of freezing and thawing, which were followed by extrusion (11 times) through two 100 nm polycarbonate membranes (Whatman Nuclepore, Sigma-Aldrich) in an Avanti Polar Lipids extrusion system (Inc. Alabaster, USA).

For the dye leakage assays, the POPC film was hydrated with a 75 mM calcein solution at pH 7.2 (20 mM HEPES buffer) containing NaCl at 150 mM before undergoing five freeze-thaw cycles and then extrusion (11 times) through membranes with pores of 100 nm diameter. The dye outside the calcein-loaded vesicles was removed by gel filtration through a Sephadex G-50 column equilibrated with a 20 mM HEPES buffer (pH 7.2) containing 150 mM NaCl.

### Circular dichroism spectroscopy

The analysis of the peptide secondary structure preferences have been performed by CD spectroscopy, for the three peptides in water and in TFE:H_2_O solutions (0:100; 10:90; 20:80; 30:70; 50:50 and 60:40 – v:v), in the presence of SDS and DPC micelles (detergent concentrations ranging from 0.01 to 20 mM), as well as in the presence of POPC and POPC:POPG (3:1 mol:mol) phospholipid vesicles (lipid concentrations ranging from 0.01 to 2.0 mM for POPC and from 0.001 to 1.0 mM for POPC:POPG 3:1). CD spectra were recorded at 20 °C on a Jasco-815 spectropolarimeter coupled to a Peltier Jasco PTC-423 L (Tokyo, Japan) using a 1.0 mm path length rectangular quartz cuvette (NSG, Farmingdale NY). All spectra were recorded from 260 to 190 nm using a 1.0 nm spectral bandwidth, 0.2 nm step resolution, 50 nm.min^−1^ scan speed, and 1 s response time. Four, six and eight accumulations were respectively performed for the peptide samples prepared in TFE:H_2_O solutions, in the presence of detergent micelles and in the presence of phospholipid vesicles. Similar experiments with the respective blank solutions were also carried out in order to allow for background subtraction. Ocellatin-LB1, −LB2 and -F1 final concentrations in the samples were 45.6, 43.3 and 39.2 mM, respectively. The spectra were analyzed using the CDPro software [28, 29].

### Dye release experiments

Calcein efflux measurements induced by peptides were performed at 37 °C on a Varian Cary Eclipse spectrofluorimeter (USA). In a typical experiment, calcein-loaded LUV solution (5 μL) was added to 2.5 mL of 150 mM NaCl and 20 mM HEPES (pH 7.2) in a quartz cuvette (NSG Precision Cells, USA) and equilibrated for some minutes at 37 °C inside the spectrofluorimeter. To induce calcein release, an aliquot of peptide solution was added to the cuvette while the sample was excited at 505 nm, and the intensity of fluorescence (*I*) was recorded at 513 nm for 6 min after which 10 μL of a Triton X-100 solution (1% v/v) was added to determine the maximum fluorescence intensity (100% leakage, *I*_max_). The percentage of calcein released from the vesicles (*I*_%_) was calculated according to the formula *I*_%_ = 100.(*I*- *I*_o_)/(*I*_max_- *I*_o_), where *I*_o_ represents the intensity of fluorescence before adding the peptide to the solution. The final peptide concentrations used in these experiments were: 3.65, 7.30, 14.66 and 21.90 μmol.mL^−1^ for ocellatin-LB1; 3.46, 6.94, 13.88 and 20.70 μmol.mL^−1^ for ocellatin-LB2; and 0.39, 0.79, 1.57, 3.14 and 6.28 μmol.mL^−1^ for ocellatin-F1.

### Antimicrobial assays

The minimum inhibitory concentration values (MICs) of the ocellatins and of the conventional antibiotic were determined by the broth microdilution susceptibility test following the guidelines of the CLSI [30, 31]. Serial dilutions of each peptide were prepared (final volume of 50 μL) in 96-well microplate with Müller-Hinton broth for bacteria and Sabouraud Dextrose agar for fungi. Each dilution series included control wells without peptide. A total of 50 μL of the adjusted inoculum (approximately 5 × 10^5^ cells/mL for bacteria or 5 × 10^3^ cells/mL for fungi, in the appropriate medium) was added to each well. To evaluate the MIC, microtiter plates with bacteria and fungi were incubated at 37 °C for 24 h.

### Hemolytic activity experiments

Rabbit blood erythrocytes (Alsever) were separated from plasma through sedimentation, suspended in a phosphate-buffered saline solution (0.14 M NaCl; 2.7 mM KCl; 10 mM Na_2_HPO_4_, 1.8 mM NaH_2_PO_4_, pH 7) and incubated with the peptides at different concentrations for 1 h at 37 °C. Erythrocytes were, then, spun down and the released hemoglobin was measured spectrophotometrically at 405 nm. An aqueous 1% v/v Triton X- 100 solution was used as a positive control for 100% of erythrocyte lysis.

## Results and discussion

**Fig. 1:**
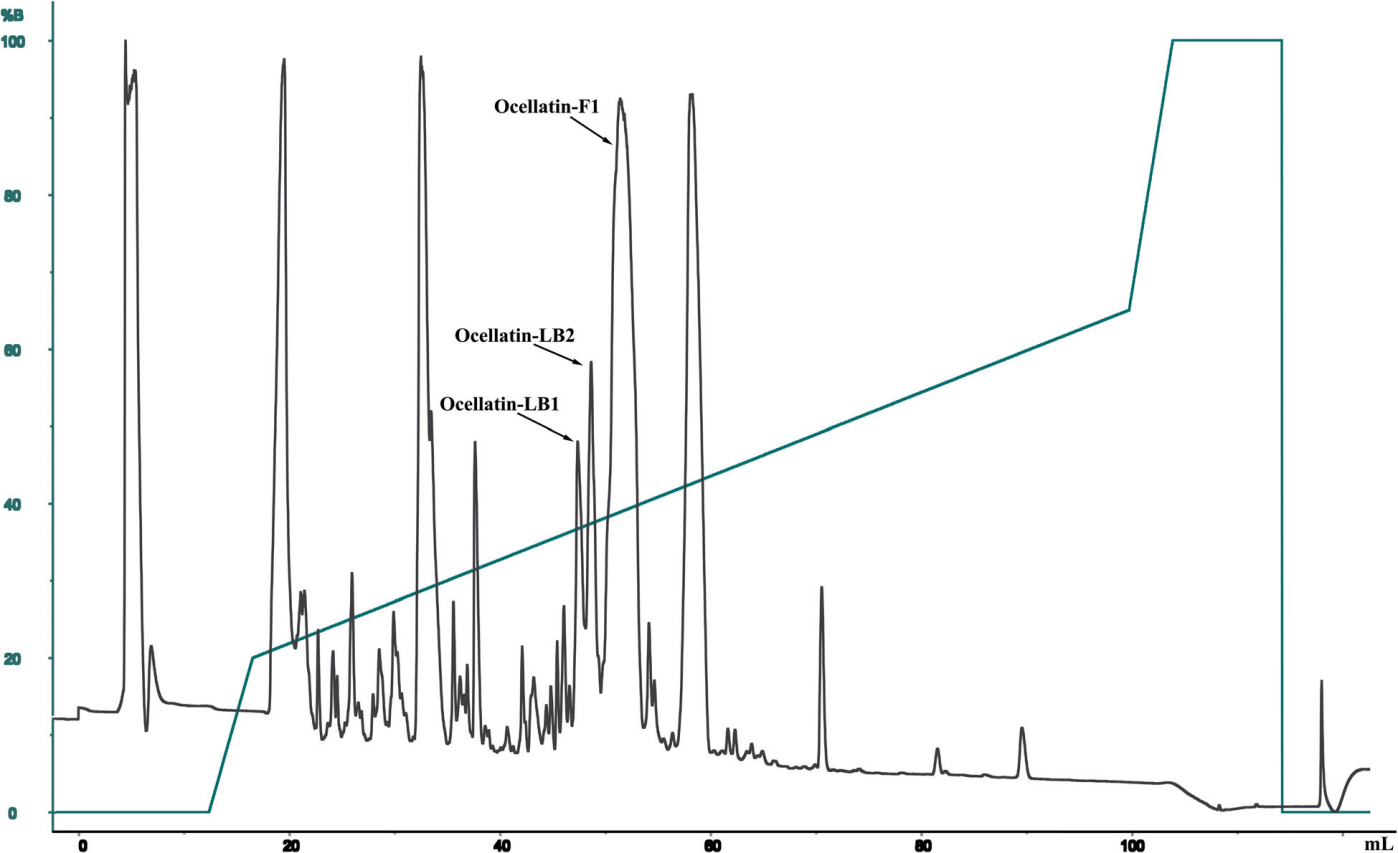
RP-HPLC profile on a preparative C8 reversed-phase column (Discovery Supelco – 4.6 × 250 mm) of pooled skin secretion of *L. labyrinthicus*. Left axis: acetonitrile concentration along the gradient. The arrow indicates the fractions that were sequenced by automated Edman degradation

**Fig. 2:**
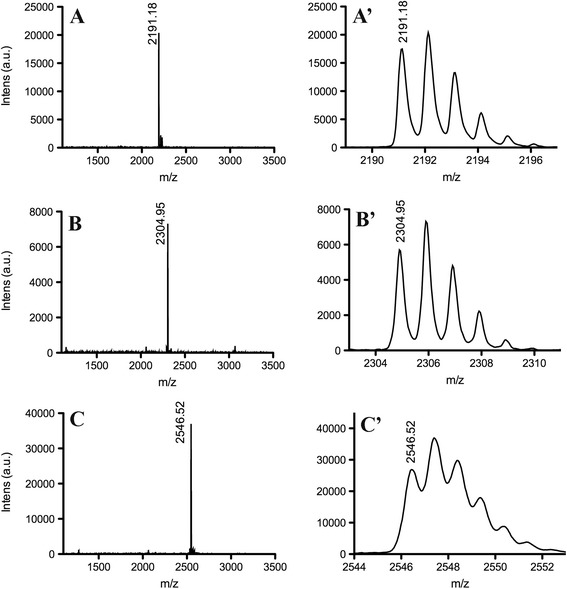
Mass spectra (MALDI-TOF-MS) and expansions of the fractions corresponding to (A, A’) ocellatin-LB1, (B, B’) ocellatin-LB2 and (C, C’) ocellatin-F1

Three peptides have been isolated from the skin secretion of *Leptodactylus labyrinthicus* (Fig. 1) and their sequences have been determined by automated Edman degradation. MALDI-TOF-TOF mass spectrometry (Fig. 2) indicated that the three peptides are naturally amidated at C-terminus and confirmed the peptide primary structures determined by Edman degradation. The primary structures of the three peptides are shown on Table 1. The three sequences present high homology, which reaches 100% for the first 22 amino acid residues, i.e., the sequences are identical from Gly-1 to Met-22, whereas ocellatin-LB2 carries and extra Asn residue and ocellatin-F1 extra Asn-Lys-Leu residues. Ocellatin-LB1 and -LB2 sequences carry three Lys and two Asp residues, which suggest a net +1 charge at physiological pH. Ocellatin-F1 carries an extra Lys residue near C-terminus (Lys-24), which implies in a net +2 charge.

**Table 1:** Primary structures of three ocellatins determined by Edman degradation

Name	Sequence	Number of residues	M_th_^a^	M_ob_^a^
ocellatin-LB1	GVVDI LKGAA KDIAG HLASK VM-NH_2_	22	2191.24	2191.18
ocellatin-LB2	GVVDI LKGAA KDIAG HLASK VMN-NH_2_	23	2305.08	2304.95
ocellatin-F1	GVVDI LKGAA KDIAG HLASK VMNKL-NH_2_	25	2546.46	2546.52

^*a*^ *M*_*th*_ and *M*_*ob*_ correspond to the theoretical monoisotopic mass and observed monoisotopic mass of the amidated peptides, respectively

Ocellatin-F1, previously known as fallaxin, was originally found in the skin secretion of *Leptodactylus fallax* and it was also recently isolated from the skin secretion *Leptodactylus labyrinthicus* [24, 26]. Whereas this peptide was active against bacteria, no activity against the tested fungal strains was observed [24]. Besides, Cunha Neto et al. [26] have noted a synergic antiviral effect between ocellatin-F1 and the alkaloid bufotenine, since combinations of these compounds lead to pronounced inhibition of BHK-21 cellular infection promoted by the rabies virus. Cunha Neto et al. [26] also mentioned the isolation of a truncated peptide sequence, which corresponds to ocellatin-F1 deprived from Lys and Leu residues at the peptide C-terminus, although no biological activity assays with this peptide have been reported up to our knowledge. This sequence was also characterized in our investigations and it corresponds to the primary structure of ocellatin-LB2 (Table 1). According to the nomenclature proposed by Conlon [32], the names ocellatin-LB1 and -LB2 were attributed to the truncated sequences.

The sequence alignment of these ocellatins with other peptides (Table 2) indicates that these compounds could present antimicrobial activities. Therefore, these three peptides have been prepared with the amidated C-terminus by synthesis on a Rink amide resin (see Additional file 1) and they were submitted to activity assays against gram-positive and gram-negative bacteria and against two fungal strains (Table 3).

**Table 2:** Amino acid sequences of antimicrobial peptides

Name	Sequence
Ocellatin-LB1	GVVDILKGAAKDIAGHLASKVM---
Ocellatin-LB2	GVVDILKGAAKDIAGHLASKVMN--
Ocellatin-F1	GVVDILKGAAKDIAGHLASKVMNKL
Ocellatin-K1	GVVDILKGAAKDLAGHLASKVMNKI
Ocellatin-S1	GVLDILKGAAKDLAGHVATKVINKI
Ocellatin-1	GVVDILKGAGKDLLAHLVGKISEKV
Ocellatin-PT3	GVIDIIKGAGKDLIAHAIGKLAEKV

Sequence alignment of ocellatin-F1, -LB1 and -LB2 against other amphipathic antimicrobial peptides. These sequences were aligned with Clustal Omega [52]. Sequences are from the following references: ocellatin-S1 [23], ocellatin-1 [53], ocellatin-K1 and ocellatin-PT3 [20]

**Table 3:** Minimal inhibitory concentrations determined for ocellatin-LB1, -LB2 and -F1 in the presence of bacteria and fungi

	MIC (μM)^a^					
Strain	ATCC	Classification	Ocellatin-LB1	Ocellatin-LB2	Ocellatin-F1	Control
*Aggregatibacter actinomycetemcomitans*	29522	Gram-negative/anaerobic	222.37	210.04	24.84	125.0^a^
*Escherichia coli*	25922	Gram-negative/aerobic	114.04	ND	397.45	125.0^a^
*Stafilococcus aureus*	25923	Gram-positive/aerobic	ND	ND	109.91	31.0^a^
*Streptococcus sanguinis*	10556	Gram-positive/anaerobic	ND	ND	ND	62.5^a^
*Candida albicans*	18804	Fungus-yeast	233.55	ND	ND	NT
*Candida lusitaniae*	56936	Fungus-yeast	233.55	ND	50.25	NT

^a^Control: chlorhexidine acetate. *ND* not determined. *NT* not tested

Among the three peptides, only ocellatin-LB1 showed activity against *Candida albicans*, however only at the highest tested concentration. Whereas ocellatin-LB2 did not presented activity against *Candida lusitaniae*, ocellatin-F1 showed pronounced activity against this fungal strain and ocellatin-LB1 showed activity only at the highest studied concentration. All of the three peptides are active against gram-negative *Aggregatibacter actinomycetemcomitans* bacteria; however, a MIC about ten times smaller is observed for ocellatin-F1 when compared to the other two ocellatins. Ocellatin-LB1 and -F1 also showed activity against gram-negative *Escherichia coli*. In the case of gram-positive bacteria, only ocellatin-F1 showed activity against the tested *Staphylococcus aureus* strain. These results suggest that ocellatin-F1 presents a stronger antibiotic potential and a broader spectrum compared to ocellatin-LB1 and -LB2, since ocellatin-F1 is active against yeast as well as gram-positive and gram-negative bacteria.

Among the three peptides, ocellatin-LB2 seems to present the smallest antimicrobial potential, since it is active only against a single tested gram-negative bacterial strain. Similarly to the observed for ocellatin-LB1 and -LB2, other AMPs such as leptoglycin and ocellatin-L1 (previously known as laticeptin), which were also isolated from the skin secretion of anurans, present restrict profiles of activities and are efficient only against gram-negative bacteria [21, 33]. According to Rollins-Smith et al. [24], the presence of high amounts of peptides in the anuran skin secretion may compensate for their relatively low antimicrobial activities, since the peptide concentration may exceed the MIC values for many pathogens to which the animal is exposed in the wild. Although the three ocellatins present activities relatively smaller when compared to other antimicrobial peptides such as DD K and LyeTx-I, they can be important to the host defense system [17, 34]. The classical screening of antimicrobial peptides is usually performed over bacterial strains pathogenic to humans; however, the production and release of peptide antibiotics from the animal skin depend on environmental and species-specific factors [21]. Contrarily to *C. albicans*, *C. lusitaniae* is a relatively rare pathogen originally isolated from the gastrointestinal tract of warm-blooded animal species, which suggests that ocellatin-F1 may be important for the animal innate immune system at its natural habitat [35, 36].

**Fig. 3:**
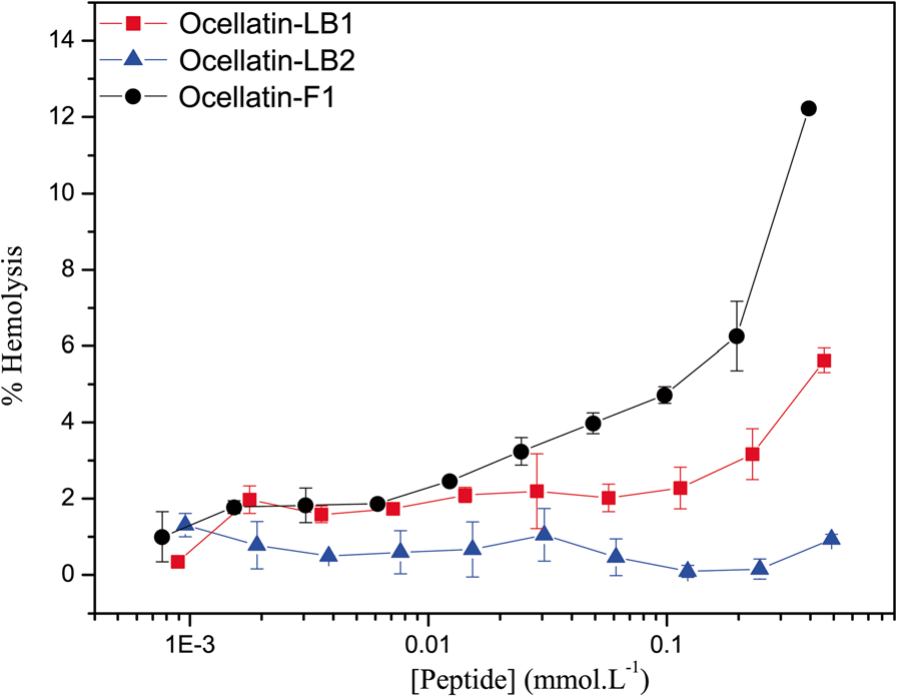
Hemolytic activities of ocellatin-LB1 (*red squares*), -LB2 (*blue triangles*) and -F1 (*black circles*). Rabbit erythrocytes suspended in phosphate-buffered saline were incubated for 1 h with increasing peptide concentrations up to 1000 μg.mL^−1^

The hemolytic effects exerted by the three ocellatins on rabbit blood erythrocytes are presented in Fig. 3. The peptides show week hemolytic activities even at the highest investigated concentrations (1000 μg.mL^−1^), which lead to hemolysis of only 6%, 1% and 13% of the cells for the assays with ocellatin-LB1, -LB2 and -F1 at 0.46, 0.50 and 0.40 μM, respectively. These values are significantly smaller when compared to the percentage of hemolysis induced by the antimicrobial peptide LyeTx-I, which promotes hemolysis of 50% of the rabbit blood erythrocytes at 0.13 μM (ED_50_) [17]. In spite of presenting low hemolytic effects, the ability of these ocellatins to promote lysis of rabbit blood erythrocytes correlates directly with their antimicrobial activities, i.e., ocellatin-F1 > ocellatin-LB1 > ocellatin-LB2. As previously mentioned, the relatively low antimicrobial activities can be balanced by peptide concentrations that exceed the MIC values and the very low hemolytic effect can afford the desired selectivity, which suggests that these compounds can be investigated as prototypes to the development of antimicrobial agents.

**Fig. 4:**
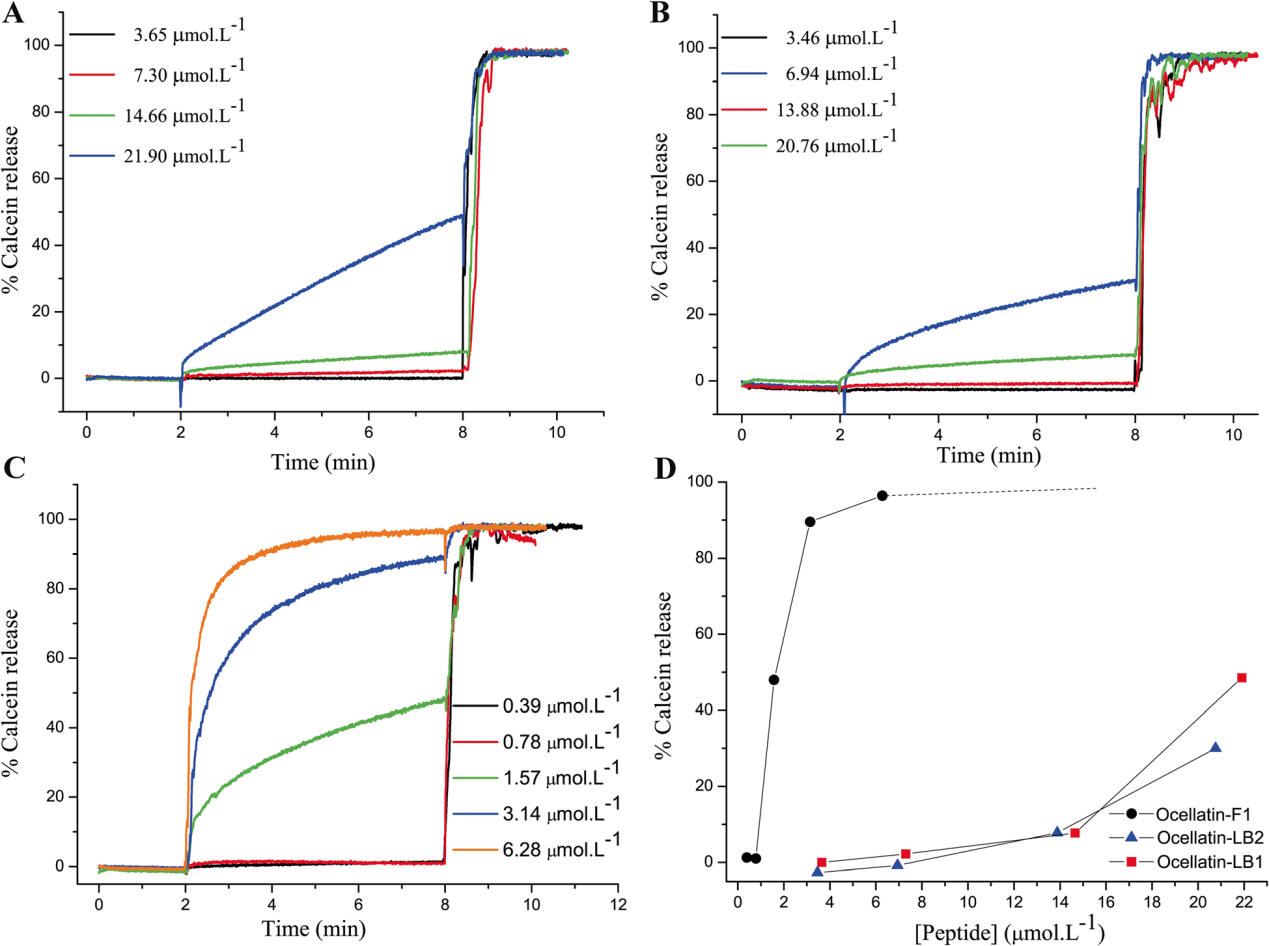
Kinetics of calcein release from POPC vesicles at 37 °C induced by different concentrations of (**a**) ocellatin-LB1, (**b**) ocellatin-LB2 and (**c**) ocellatin-F1. The vesicle solutions were equilibrated for 2 minutes at 37 °C inside the spectrofluorimeter before the addition of peptide. **d** Maximum percentage of calcein release as a function of the peptide concentration for ocellatin-LB1 (*red squares*), -LB2 (*blue triangles*) and -F1 (*black circles*)

The membrane-disruptive properties of the three sequenced ocellatins were investigated by assays of dye leakage promoted by the peptides on calcein-loaded POPC vesicles and the obtained results indicate that ocellatin-F1 interacts differently with POPC vesicles, when compared to the other two ocellatins (Fig. 4). Ocellatin-F1 at 1.57 mM is able to promote a dye release of 48%, on the other hand ocellatin-LB1 and -LB2 at concentrations near to 7 mM promote dye releases no greater than 2.16%. Ocellatin-F1 at a similar concentration (6.28 mM) is able to promote 96% of dye release; however, peptide concentrations near to 20 mM promote maximum dye releases of only 48.5% and 30% for ocellatin-LB1 and -LB2, respectively. Whereas ocellatin-F1 presents pronounced disruptive properties even at very low concentrations, the dye leakage induced by ocellatin-LB1 and -LB2 seems to be more dose-dependent, which is characteristic of a cooperative mechanism that seems to demand the accumulation of peptides on the bilayer surface to effectively promote the membrane lysis [37]. These distinct mechanisms may be advantageous to the animal, since a larger choice implies in a defense mechanism that is efficient against different pathogens [38–40]. The conjoint action of these mechanisms promoted by the simultaneous secretion of different peptides can even lead to a more robust defense system [41, 42]. Interestingly the pore formation capacities of the three ocellatins correlate directly to their antimicrobial and hemolytic activities, i.e., ocellatin-F1 > ocellatin-LB1 > ocellatin-LB2.

**Fig. 5:**
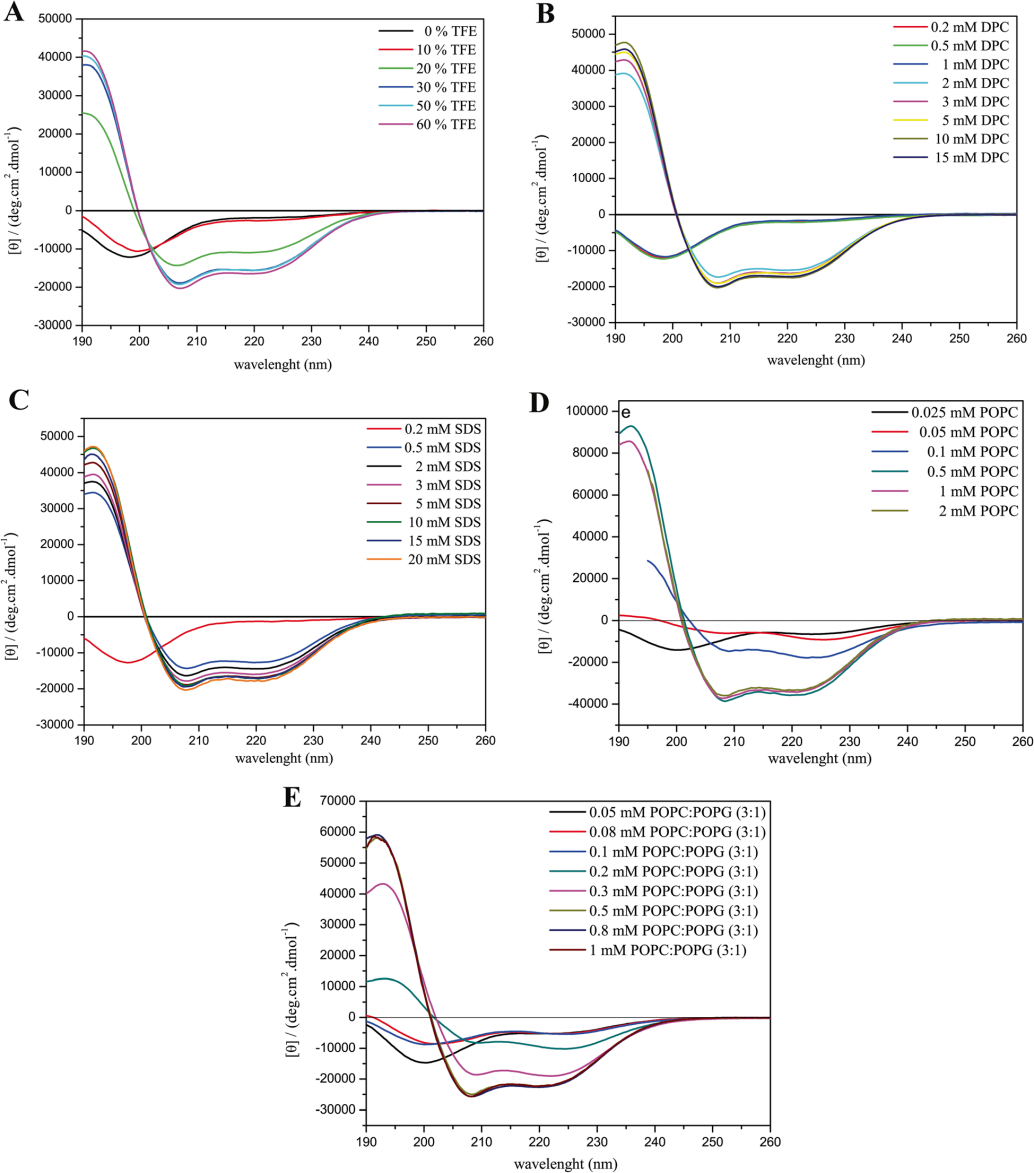
CD spectra of ocellatin-LB1 in the presence of (**a**) TFE:H_2_O solutions, (**b**) DPC and (**c**) SDS micelles, (**d**) POPC and (**e**) POPC:POPG (3:1) vesicles

**Fig. 6:**
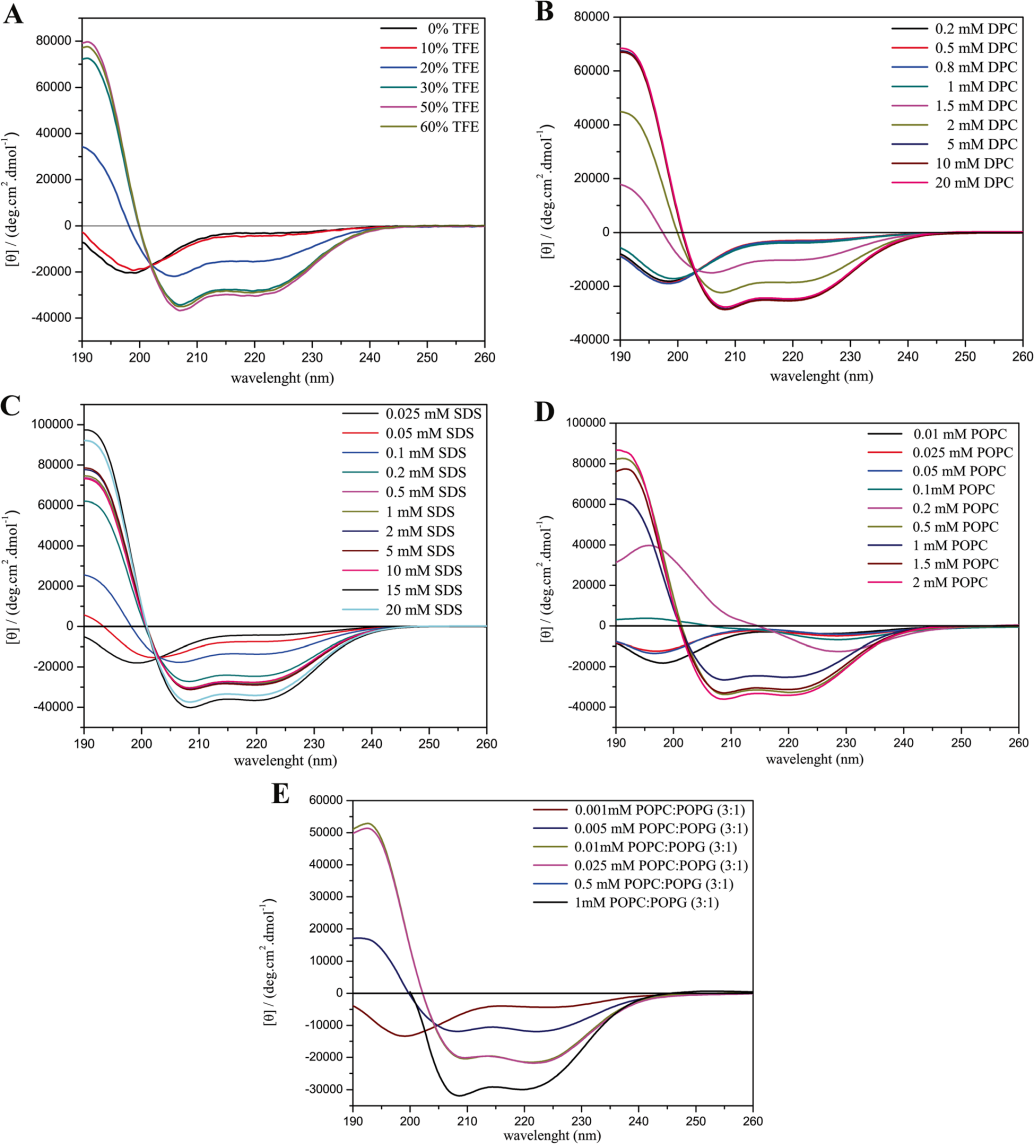
CD spectra of ocellatin-LB2 in the presence of (**a**) TFE:H_2_O solutions, (**b**) DPC and (**c**) SDS micelles, (**d**) POPC and (**e**) POPC:POPG (3:1) vesicles

**Fig. 7:**
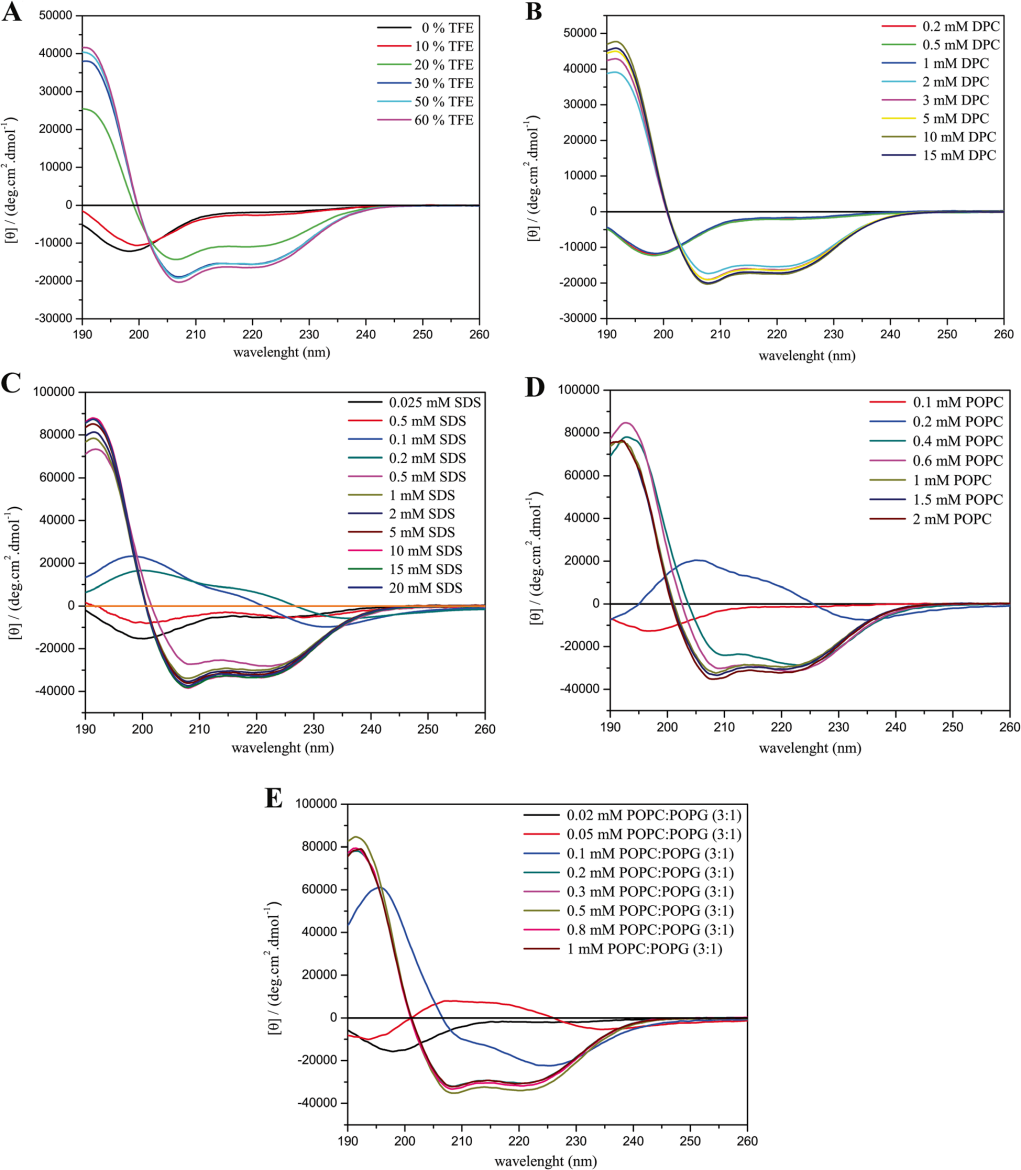
CD spectra of ocellatin-F1 in the presence of (**a**) TFE:H_2_O solutions, (**b**) DPC and (**c**) SDS micelles, (**d**) POPC and (**e**) POPC:POPG (3:1) vesicles

**Fig. 8:**
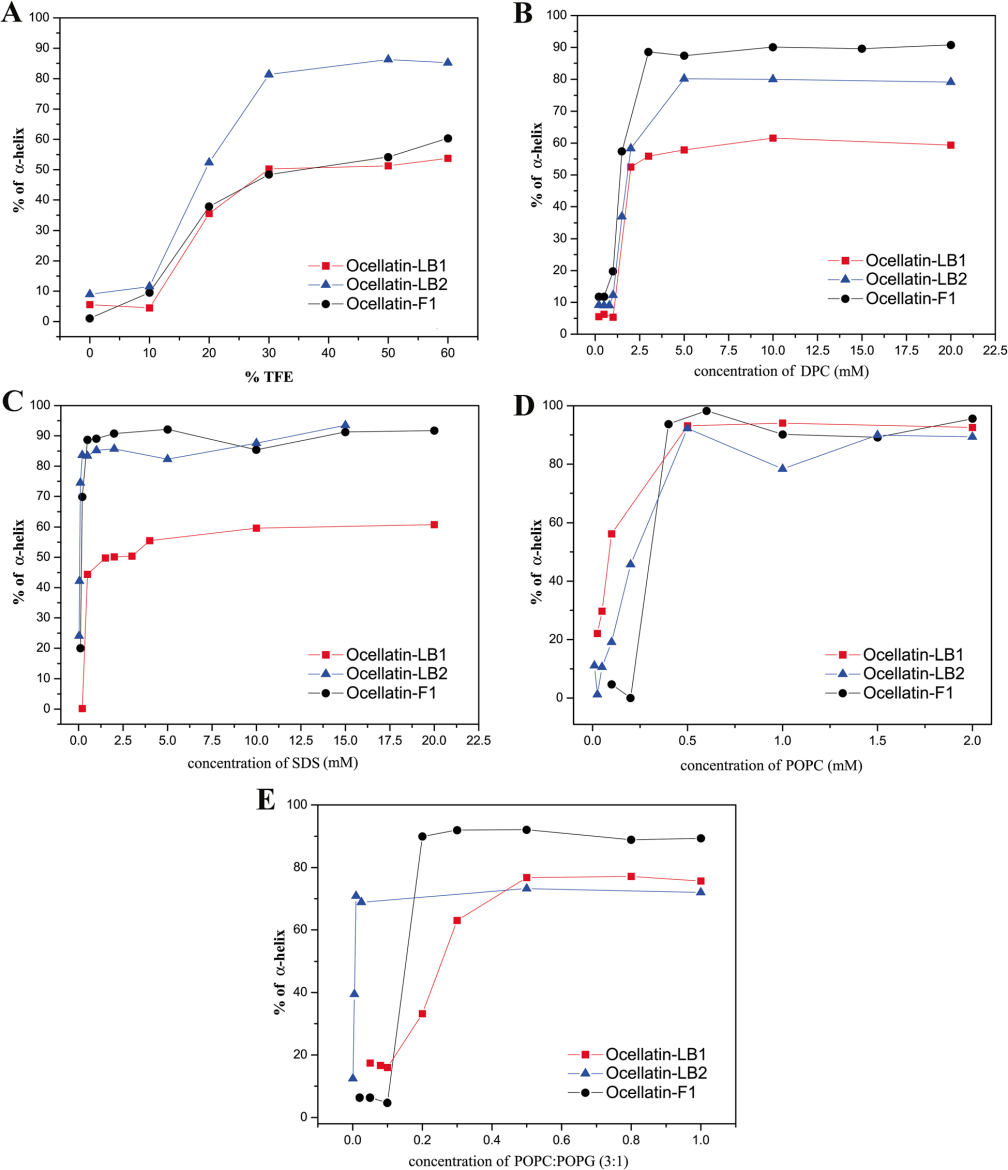
Helical contents of ocellatin-LB1 (*red squares*), -LB2 (*blue triangles*) and -F1 (*black circles*) in the presence of (**a**) TFE:H_2_O solutions and as a function of (**b**) DPC, (**c**) SDS, (**d**) POPC and (**e**) POPC:POPG (3:1) concentrations

The CD spectra obtained for the three ocellatins in several media are presented in Figs. 5, 6 and 7 and the respective percentage of helical contents evaluated from spectral deconvolution is summarized in Fig. 8. In aqueous media (panel A) all of the peptides present spectra which are consistent with random coil conformations, as evidenced by a characteristic minimum at 198 nm. In the presence of 10% of TFE, it is possible to observe a positive shift of the minimum, however the spectral profiles undergo significant modifications only at 20% of TFE, where two minima are observed near 208 and 222 nm. At higher proportions of TFE it is observed an enhancement of these two minima, which is consistent with well-defined helical segments. This behavior is typical of linear antimicrobial peptides, which usually do not present conformational preferences in water, but well-defined active conformations are acquired when they reach the membrane surface [43–45].

Similar behaviors are observed for the peptides in the presence of zwitterionic (panel B) and anionic (panel C) detergent micelles and a well-defined maximum near 193 nm is observed for SDS concentrations as small as 0.5 mM and for DPC concentrations as small as 2.0 mM. In the presence of both micellar solutions, ocellatin-LB1 presents smaller helical contents than the other two ocellatins, whereas in the presence of DPC micelles, ocellatin-F1 presents a higher helicity even when compared to ocellatin-LB2. The spectra obtained for the peptides in the presence of zwitterionic (POPC) and anionic (POPC:POPG 3:1) phospholipid bilayers are also consistent with helical profiles (panels D and E, respectively). However, greater enhancements of the minimum and the maximum intensities suggest that the three peptides acquire even higher helical contents in the presence of phospholipid vesicles, when compared to the peptides in the presence of TFE:H_2_O or aqueous micellar solutions. Helicity higher than 90% is reached for all the peptides at higher concentrations of POPC, although ocellatin-F1 seems to present slightly higher helical contents in comparison with the other two peptides.

Interestingly, in the presence of anionic POPC:POPG (3:1) vesicles, ocellatin-F1 shows significantly stronger helical propensities in comparison to ocellatin-LB1 and -LB2. Whereas ocellatin-F1 presents similar helical contents in both membranes, ocellatin-LB1 and -LB2 clearly present larger helical segments in the presence of POPC bilayers. This behavior is somehow unusual to cationic peptides, which show more often stronger affinities for negatively charged membranes [46, 47]. However, in the case of these three ocellatins, despite the net positive charges, the presence of two negatively charged aspartate residues may somehow modulate the membrane binding process, due to the possibility of some repulsive interaction between these residues and the negative POPG lipid head groups. This may explain the apparent stronger affinity of ocellatin-LB1 and -LB2 to the zwitterionic vesicles; however, the determination of the detailed three dimensional structures is required for confirming this assumption. Another point that reinforces this proposal is that the extra positive charge of ocellatin-F1 creates very high structural order in the presence of the anionic bilayers, possibly by either interacting more efficiently with the negative lipid head groups, or by neutralizing some repulsive effect exerted by one of the aspartate residues.

An important aspect is that the minimum and the maximum intensities observed in the CD spectra of the peptides in the presence of phospholipid vesicles are significantly greater that the ones observed in the presence of TFE:H_2_O solutions (Figs. 5, 6 and 7), which is confirmed by deconvolution of the respective spectra (Fig. 8). These results are very representative, since, in several cases, antimicrobial peptides that partition between membrane and aqueous environments usually present higher helicities in the presence of TFE:H_2_O mixtures, whereas moderate or small structural contents are observed in the presence of phospholipid vesicles, due to simultaneous contributions of aqueous random-coil and structured membrane-bound populations [13, 48]. In the presence of POPC, bilayers helicities higher than 90% are observed for the three peptides, whereas in TFE:H_2_O solutions helical contents of only 53.7, 85.2 and 60.4% have been observed for ocellatin-LB1, -LB2, and -F1, respectively (Fig. 8). Therefore, contrarily to several reported cases [9, 40, 48], significantly higher structural degrees are observed for the peptides in the presence of vesicles and these results indicate that these three ocellatins show high affinity for the phospholipid bilayers.

It is known that fragments derived from active peptides can be found in the skin secretion of frogs and, in particular, it was shown that the crude skin secretion of *Leptodactylus labyrinthicus* is rich in metallo and serine peptidases [49]. The peptide sequences investigated here present 100% homology from residues 1 to 22 (ocellatin-LB1 primary structure), ocellatin-LB2 carries an extra Asn and ocellatin-F1 extra Asn-Lys-Leu residues. Therefore, it is possible that enzymes involved in proteolytic cleavages are related to the production of peptide segments. However, regardless of the biochemical processes responsible for the peptide processing, from the chemical synthesis point of view, ocellatin-LB1 can be considered as a template for this series and the extra amino acid residues present in ocellatin-LB2 and -F1 sequences seem to have important effects on their biological activities, membrane-disruptive properties and secondary structure profiles.

Ocellatin-F1 presents a stronger antibiotic potential and a broader spectrum of activities compared to the other two peptides, whereas ocellatin-LB2 presents a smaller antimicrobial potential in comparison with the chemical template ocellatin-LB1. Although the three peptides showed low hemolytic activity, they correlated directly with their antimicrobial potential, i.e., ocellatin-F1 > ocellatin-LB1 > ocellatin-LB2. Dye release assays performed for the three peptides with solutions containing calcein-loaded phospholipid vesicles also indicated that ocellatin-F1 pore formation activity is significantly stronger than that of ocellatin-LB1, which is more effective than ocellatin-LB2 in disrupting the bilayer integrity.

Although CD spectroscopy indicated that the three peptides show high helical contents in the presence of detergent micelles as well as in the presence of phospholipid bilayers, it is clear that ocellatin-F1 in average presents a higher helical propensity in comparison with the other two peptides. Therefore, the extra three amino acid residues present in the sequence of ocellatin-F1 seem to assure stronger membrane-interactions for ocellatin-F1 in comparison to the other two ocellatins. This effect is very likely to be related to the presence of the Lys residue near ocellatin-F1 C-terminus (Lys-24), which may promote more efficient dipole neutralization for ocellatin-F1 as well as assure more efficient electrostatic interactions with the membrane.

These results altogether indicate that the three extra residues present at the ocellatin-F1 C-terminus play an important role in promoting stronger peptide-membrane interactions and antimicrobial properties, therefore the C-terminus of ocellatin-F1 seems to be extremely important for the peptide activity. Interestingly, the extra Asn-23 residue present in ocellatin-LB2 sequence seems to decrease its antimicrobial potential and the strength of the peptide-membrane interactions in comparison to ocellatin-LB1. In this sense, it seems to be worth in future investigations to promote site-directed substitutions at Asn-23 in ocellatin-F1 sequence in order to improve the biological activities of this peptide series [50]. Naturally, structural and topological information obtained from other biophysical approaches, such as solution and solid-state NMR spectroscopies [46, 51], can be used to gain information about the peptide-membrane interaction process and may give important insights about the amino acid substitutions at position 23, which can easily be performed by solid-phase peptide synthesis.

## Conclusions

Despite the high sequence homology of the three investigated peptides present in the skin secretion of *Leptodactylus labyrinthicus*, these compounds show distinct antimicrobial spectra as well as different hemolytic activities and membrane-disruptive properties. The stronger antimicrobial properties of ocellatin-F1 correlate directly with its stronger membrane interactions, higher helical propensities and pore formation capacity, when compared to ocellatin-LB1 and -LB2. Whereas the extra Asn-Lys-Leu residues present at ocellatin-F1 C-terminus (positions 23 to 25) seem to promote stronger peptide-membrane interactions and higher antimicrobial activities, the extra Asn-23 residue of ocellatin-LB2 seems to decrease its antimicrobial potential.

## Additional file

Additional file 1: Analytical HPLC profiles and mass spectra (MALDI-TOF-MS) of the purified synthetic peptides (A, A’) ocellatin-LB1, (B, B’) ocellatin-LB2 and (C, C’) ocellatin-F1. The samples were injected (200 μL solution at 1 mg.mL^−1^) into a C18 Vydac 218TP510 column (250 mm × 10 mm) equilibrated with 0.1% TFA. Elution: acetonitrile solution/0.1% TFA at a flow rate of 1 mL.min^−1^. The straight line depicts the variation of the acetonitrile concentration. Absorbance was monitored (λm) at 214 nm. (JPG 374 kb)

## Abbreviations

AMPs: Antimicrobial peptides
ATCC: American type culture collection
CD: Circular dichroism
CFU: Colony forming units
CHCA: α-cyano-4-hidroxycinnamic acid
CLSI: Clinical and Laboratory Standards Institute
DHB: 2,5-dihydroxybenzoic acid
DPC: Dodecylphosphocholine
HEPES: 4-(2-hydroxyethyl)piperazin-1-ethanesulfonic acid
LMVs: Large multilamellar vesicles
LUVs: Large unilamellar vesicles
MIC: Minimum inhibitory concentration
ND: Not determined
NT: Not tested
POPC: 1-palmitoyl-2-oleoyl-*sn*-glycero-3-phosphocholine
POPG: 1-palmitoyl-2-oleoyl-phosphatidylglycerol
SDS: Sodium dodecyl sulphate
TFA: Trifluoroacetic acid
TFE: 2,2,2-trifluoroethanol

## References

[1] Lazarev VN, Govorun VM (2010). Antimicrobial peptides and their use in medicine. applied biochemistry and microbiology. Appl Biochem Microbiol.

[2] Kang SJ, Kim DH, Mishig-Ochir T, Lee BJ (2012). Antimicrobial peptides: their physicochemical properties and therapeutic application. Arch Pharm Res.

[3] Wang G, Li X, Wang Z (2016). APD3: the antimicrobial peptide database as a tool for research and education. Nucleic Acids Res.

[4] Xu X, Lai R (2015). The chemistry and biological activities of peptides from amphibian skin secretions. Chem Rev.

[5] Bowie JH, Separovic F, Tyler MJ (2012). Host-defense peptides of Australian anurans. Part 2. Structure, activity, mechanism of action, and evolutionary significance. Peptides.

[6] Apponyi MA, Pukala TL, Brinkworth CS, Maselli VM, Bowie JH, Tyler MJ (2004). Host-defence peptides of Australian anurans: structure, mechanism of action and evolutionary significance. Peptides.

[7] Nascimento AC, Fontes W, Sebben A, Castro MS (2003). Antimicrobial peptides from anurans skin secretions. Protein Pept Lett.

[8] Schmidtchen A, Pasupuleti M, Malmsten M (2014). Effect of hydrophobic modifications in antimicrobial peptides. Adv Colloid Interface Sci.

[9] Sugawara M, Resende JM, Moraes CM, Marquette A, Chich JF, Metz-Boutigue MH (2010). Membrane structure and interactions of human catestatin by multidimensional solution and solid-state NMR spectroscopy. FASEB J.

[10] Hale JD, Hancock RE (2007). Alternative mechanisms of action of cationic antimicrobial peptides on bacteria. Expert Rev Anti Infect Ther.

[11] Brogden KA (2005). Antimicrobial peptides: pore formers or metabolic inhibitors in bacteria?. Nat Rev Microbiol.

[12] Bürck J, Wadhwani P, Fanghänel S, Ulrich AS (2016). Oriented circular dichroism: a method to characterize membrane-active peptides in oriented lipids bilayers. Acc Chem Res.

[13] Voievoda N, Schulthess T, Bechinger B, Seelig J (2015). Thermodynamic and biophysical analysis of the membrane-association of a histidine-rich peptide with efficient antimicrobial and transfection activities. J Phys Chem B.

[14] Avitabile C, D'Andrea LD, Romanelli A (2014). Circular dichroism studies on the interactions of antimicrobial peptides with bacterial cells. Sci Rep.

[15] Bechinger B, Resende JM, Aisenbrey C (2011). The structural and topological analysis of membrane-associated polypeptides by oriented solid-state NMR spectroscopy: established concepts and novel developments. Biophys Chem.

[16] Verly RM, Rodrigues MA, Daghastanli KR, Denadai AM, Cuccovia IM, Bloch CJ (2008). Effect of cholesterol on the interaction of the amphibian antimicrobial peptide DD K with liposomes. Peptides.

[17] Santos DM, Verly RM, Piló-Veloso D, de Maria M, de Carvalho MA, Cisalpino PS (2010). LyeTx I, a potent antimicrobial peptide from the venom of the spider *Lycosa erythrognata*. Amino Acids.

[18] Azevedo Calderon L, Silva AA, Ciancaglini P, Stábeli RG (2011). Antimicrobial peptides from *Phyllomedusa* frogs: from biomolecular diversity to potential nanotechnologic medical applications. Amino Acids.

[19] Frost D. Amphibian Species of the World: an Online Reference. Version 5.6. 2013. Electronic. Electronic Database accessible at http://research.amnh.org/herpetology/amphibia/index.html. American Museum of Natural History, New York, USA. Accessed 15 Sept 2016.

[20] Marani MM, Dourado FS, Quelemes PV, de Araujo AR, Perfeito ML, Barbora EA (2015). Characterization and biological activities of Ocellatin peptide from the skin secretion of the frog *Leptodactylus pustulatus*. J Nat Prod.

[21] Sousa JC, Berto RF, Gois EA, Fontenele-Cardi NC, Honório JE, Konno K (2009). Leptoglycin: a new Glycine/Leucine-rich antimicrobial peptide isolated from the skin secretion of the South American frog *Leptodactylus pentadactylus* (Leptodactylidae). Toxicon.

[22] King JD, Rollins-Smith LA, Nielsen PF, John A, Conlon JM (2005). Characterization of a peptide from skin secretions of male specimens of the frog, *Leptodactylus fallax* that stimulates aggression in male frogs. Peptides.

[23] Dourado FS, Leite JR, Silva LP, Melo JA, Bloch C, Schwartz EF (2007). Antimicrobial peptide from the skin secretion of the frog *Leptodactylus syphax*. Toxicon.

[24] Rollins-Smith LA, King JD, Nielsen PF, Sonnevend A, Conlon JM (2005). An antimicrobial peptide from the skin secretions of the mountain chicken frog *Leptodactylus fallax* (Anura: Leptodactylidae). Regul Pept.

[25] Libério MS, Joanitti GA, Azevedo RB, Cilli EM, Zanotta LC, Nascimentos AC (2011). Anti-proliferative and cytotoxic activity of pentadactylin isolated from *Leptodactylus labyrinthícus* on melanoma cells. Amino Acids.

[26] Neto RSC, Vigerelli H, Jared C, Antoniazzi MM, Chaves LB, da Silva ACR (2015). Synergic effects between ocellatin-F1 and bufotenine on the inhibition of BHK-21 cellular infection by the rabies virus. J Venom Anim Toxins incl Trop Dis.

[27] Chan WC, White PD (2000). Fmoc solid phase peptide synthesis.

[28] Sreerama N, Woody RW (2000). Estimation of protein secondary structure from circular dichroism spectra: comparison of CONTIN, SELCON, and CDSSTR methods with an expanded reference set. Anal Biochem.

[29] Sreerama N, Woody RW (2004). On the analysis of membrane protein circular dichroism spectra. Protein Sci.

[30] Clinical and Laboratory Standards Institute. Performance standards for antimicrobial disk susceptibility tests, approved standard, 7th ed., CLSI document M02-A11. Wayne: Clinical and Laboratory Standards Institute; 2012. http://shop.clsi.org/site/Sample_pdf/M02A12_sample.pdf

[31] Clinical and Laboratory Standards Institute. Method for antifungal disk diffusion susceptibility testing of yeasts, Approved Guideline. CLSI document M44-A2. Wayne: Clinical and Laboratory Standards Institute; 2004. http://shop.clsi.org/site/Sample_pdf/M44A2_sample.pdf

[32] Conlon JM (2008). A proposed nomenclature for antimicrobial peptides from frogs of the genus *Leptodactylus*. Peptides.

[33] Conlon JM, Al-Ghaferi N, Abraham B, Sonnevend A, King JD, Nielsen PF (2006). Purification and properties of Laticeptin, an antimicrobial peptide from skin secretions of the South American frog *Leptodactylus laticeps*. Protein Pept Lett.

[34] Batista CVF, da Silva LR, Sebben A, Scaloni A, Ferrara L, Paiva GR (1999). Antimicrobial peptides from the Brazilian frog *Phyllomedusa distincta*. Peptides.

[35] Marr KA, Seidel K, White TC, Bowden RA (2000). Candidemia in allogeneic blood and marrow transplant recipients: evolution of risk factors after the adoption of prophylactic fluconazole. J Infect Dis.

[36] Merz WG (1984). *Candida Lusitaniae*: frequency of recovery, colonization, infection, and amphotericin B resistance. J Clin Microbiol.

[37] Takeuchi K, Takahashi H, Sugai M, Iwai H, Kohno T, Sekimizu K (2004). Channel-forming membrane permeabilization by an antibacterial protein, sapecin: determination of membrane-buried and oligomerization surfaces by NMR. J Biol Chem.

[38] Giovannini MG, Poulter L, Gibson BW, Williams DH (1987). Biosynthesis and degradation of peptides derived from *Xenopus laevis* prohormones. Biochem J.

[39] Leite JR, Silva LP, Rodrigues MI, Prates MV, Brand GD, Lavaca BM (2005). Phylloseptins: a novel class of anti-bacterial and anti-protozoan peptides from the *Phyllomedusa* genus. Peptides.

[40] Resende JM, Moraes CM, Prates MV, Cesar A, Almeida FCL, Mundim NCCR (2008). Solutions NMR structures of the antimicrobial peptides phylloseptin-1, −2 and −3 and biological activity: the role of charges and hydrogen bonding interactions in stabilizing helix conformations. Peptides.

[41] Westerhoff HV, Zasloff M, Rosner JL, Hendler RW, De Waal A, Vaz Gomes A (1995). Functional synergism of the magainins PGLa and magainin-2 in *Escherichia coli*, tumor cells and liposomes. Eur J Biochem.

[42] Hara T, Mitani Y, Tanaka K, Uematsu N, Takakura A, Tachi T (2001). Heterodimer formation between the antimicrobial peptides magainin 2 and PGLa in lipid bilayers: a cross-linking study. Biochemistry.

[43] Bechinger B, Lohner K (2006). Detergent-like actions of linear amphipathic cationic antimicrobial peptides. Biochim Biophys Acta.

[44] Strandberg E, Ulrich AS (2004). NMR methods for studying membrane-active antimicrobial peptides. Concepts Magn Reson Part A.

[45] Bechinger B (1999). The structure, dynamics and orientation of antimicrobial peptides in membranes by multidimensional solid-state NMR spectroscopy. Biochim Biophys Acta.

[46] Resende JM, Verly RM, Aisenbrey C, Cesar A, Bertani P, Piló-Veloso D (2014). Membrane interactions of phylloseptin-1, −2, and −3 peptides by oriented solid-state NMR spectroscopy. Biophys J.

[47] Oren Z, Shai Y (2000). Cyclization of a cytolytic amphipathic α-helical peptide and its diastereomer: effect on structure, interaction with model membranes, and biological function. Biochemistry.

[48] Ladokhin AS, Fernández-Vidal M, White SH (2010). CD spectroscopy of peptides and proteins bound to large unilamellar vesicles. J Membr Biol.

[49] Libério MS, Bastos IMD, Pires Júnior OR, Fontes W, Santana JM, Castro MS (2014). The crude skin secretion of the pepper frog *Leptodactylus labyrinthicus* is rich in metallo and serine peptidases. PLoS One.

[50] Silva CN, Nunes KP, Torres FS, Cassoli JS, Santos DM, Almeida FM (2005). PnPP-19, a synthetic and nontoxic peptide designed from a *Phoneutria nigriventer* toxin, potentiates erectile function via NO/cGMP. J Urol.

[51] Verly RM, de Moraes CM, Resende JM, Aisenbrev C, Bemquerer MP, Piló-Veloso D (2009). Structure and membrane interactions of the antibiotic peptide dermadistinctin K by multidimensional solution and oriented ^15^N and ^31^P solid-state NMR spectroscopy. Biophys J.

[52] Sievers F, Wilm A, Dineen D, Gibson TJ, Karplus K, Li W (2011). Fast, scalable generation of high-quality protein multiple sequence alignments using Clustal Omega. Mol Syst Biol.

[53] Nascimento AC, Zanotta LC, Kyaw CM, Schwartz EN, Schwartz CA, Sebben A (2004). Ocellatins: new antimicrobial peptides from the skin secretion os the South American frog *Leptodactylus ocellatus* (Anura: *Leptodactylidae*). Protein J.

