# Supplementary figures and images for: Ocellatin peptides from the skin secretion of the South American frog Leptodactylus labyrinthicus (Leptodactylidae): characterization, antimicrobial activities and membrane interactions
Source: J Venom Anim Toxins Incl Trop Dis. 2017 Jan 19;23:4. doi: 10.1186/s40409-017-0094-y (PMC5244724; doi:10.1186/s40409-017-0094-y)

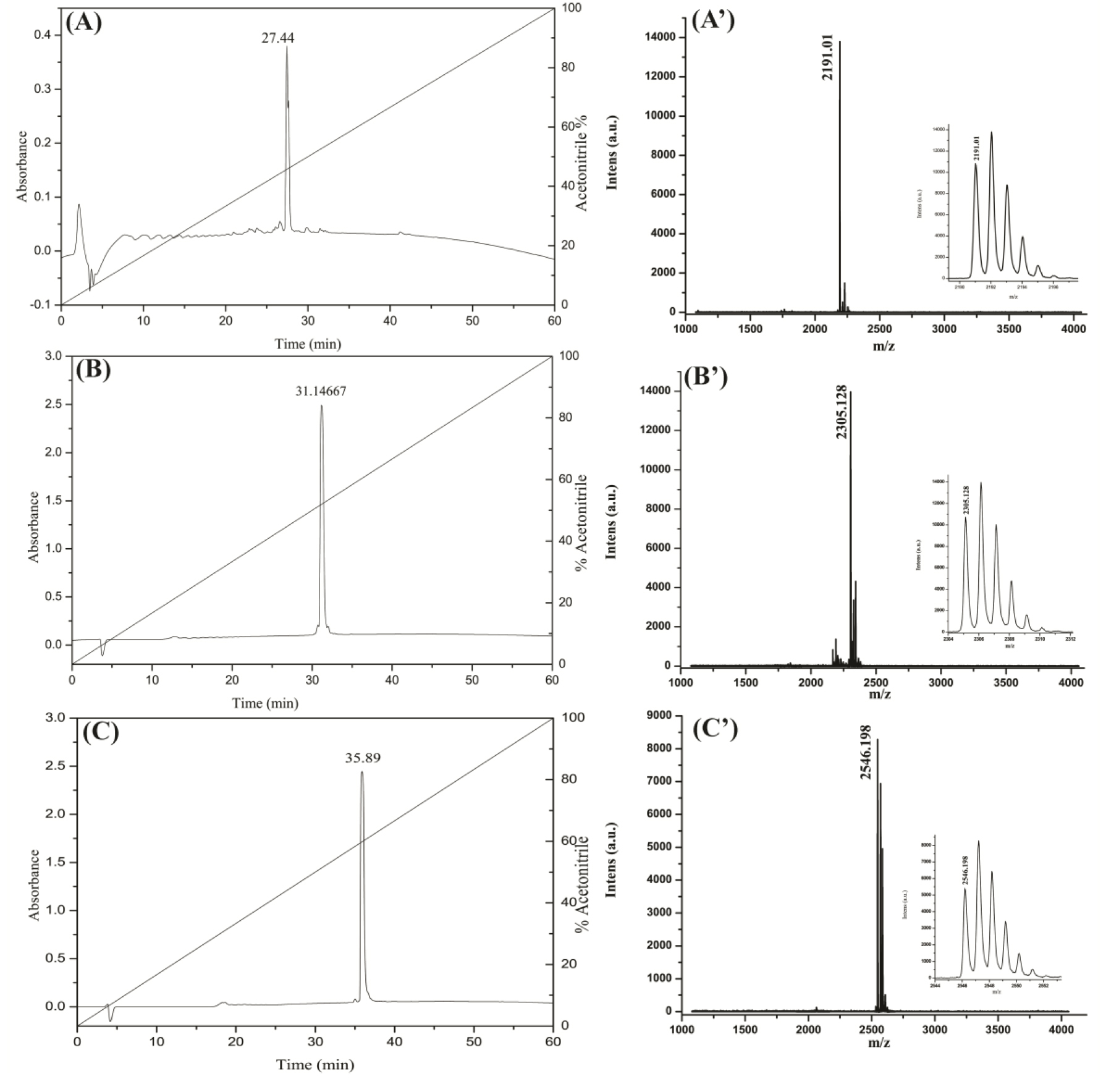

Supplement: Additional file 1: — Analytical HPLC profiles and mass spectra (MALDI-TOF-MS) of the purified synthetic peptides (A, A’) ocellatin-LB1, (B, B’) ocellatin-LB2 and (C, C’) ocellatin-F1. The samples were injected (200 μL solution at 1 mg.mL−1) into a C18 Vydac 218TP510 column (250 mm × 10 mm) equilibrated with 0.1% TFA. Elution: acetonitrile solution/0.1% TFA at a flow rate of 1 mL.min−1. The straight line depicts the variation of the acetonitrile concentration. Absorbance was monitored (λm) at 214 nm. (JPG 374 kb) [file 40409_2017_94_MOESM1_ESM.jpg]
